# Dasatinib promotes TRAIL‐mediated apoptosis by upregulating CHOP‐dependent death receptor 5 in gastric cancer

**DOI:** 10.1002/2211-5463.12404

**Published:** 2018-03-23

**Authors:** Xiaona Wang, Qiang Xue, Liangliang Wu, Baogui Wang, Han Liang

**Affiliations:** ^1^ Department of Gastric Cancer Tianjin Medical University Cancer Institute and Hospital National Clinical Research Center for Cancer Key Laboratory of Cancer Prevention and Therapy Tianjin's Clinical Research Center for Cancer China

**Keywords:** apoptosis, dasatinib, death receptor 5, gastric cancer, TRAIL

## Abstract

Dasatinib, a tyrosine kinase inhibitor, has been approved for first‐line treatment of leukemia and has also been evaluated for use in numerous other cancers. However, its role in gastric cancer (GC) remains unclear. Therefore, the aim of this study was to investigate how dasatinib suppresses the growth of GC cells and interacts with chemotherapeutic drugs. The results showed that, in the presence of dasatinib, proliferation of GC cells decreased and apoptosis increased, and that Fas‐associated death domain protein and caspase‐8 are essential to dasatinib‐induced cell apoptosis in GC. In addition, we found that dasatinib increased the expression of death receptor 5 (DR5) in GC cells. Dasatinib enhanced apoptosis induced by tumor necrosis factor‐related apoptosis‐inducing ligand (TRAIL) in GC cells. Moreover, increased DR5 expression facilitated dasatinib‐induced apoptosis; the dasatinib‐induced increase in DR5 expression was mediated by CCAAT/enhancer‐binding protein homologous protein (CHOP). Furthermore, dasatinib also synergized with TRAIL to induce apoptosis via DR5 in GC cells. Our results show that dasatinib promoted TRAIL‐mediated apoptosis via upregulation of CHOP‐dependent DR5 expression in GC, suggesting that DR5 induction can be used as an indicator of dasatinib sensitivity.

**Abbreviations**
ChIP: chromatin immunoprecipitation
CHOP: CCAAT/enhancer‐binding protein homologous protein
DR: death receptor
ER: endoplasmic reticulum
FADD: Fas‐associated death domain protein
GC: gastric cancer
shRNA: short hairpin RNA
siRNA: small interfering RNA
TNF: tumor necrosis factor
TRAIL: tumor necrosis factor‐related apoptosis‐inducing ligand
TUNEL: terminal deoxynucleotidyl transferase‐mediated dUTP nick end labeling
z‐VAD‐fmk: carbobenzoxy‐valyl‐alanyl‐aspartyl‐[*O*‐methyl]‐fluoromethylketone

Gastric cancer (GC) is the third leading cause of cancer‐associated deaths and the fifth most common malignancy worldwide; its development and progression are considered to be multistep processes, which are thought to be affected by accumulated mutations in related genes 1, 2, 3. The diagnosis of patients with GC is often delayed, which affects the prognosis 4. Thus, in‐depth research on GC development and progression is required to identify new therapeutic targets to improve GC prognosis 5.

Intrinsic and extrinsic pathways are considered to be involved in apoptosis regulation 6, 7. The mitochondrial pathway is one of the intrinsic pathways activated by Bcl‐2. Antagonized by the BH3‐only protein, Bcl‐2 can be inactivated, which in turn can induce the activation of Bax/Bak, thereby interfering with the functions of the mitochondrial outer membrane 8. The extrinsic pathway is activated by a combination of the ligands and the receptors of tumor necrosis factor (TNF), and its activity is negatively regulated by decoy receptors. TNF‐related apoptosis‐inducing ligand (TRAIL) is a ligand that induces apoptosis of cancer cells with no adverse effect on normal cells 9, 10. TRAIL can bind with death receptor (DR) 5 or DR4 on the cell surface, thereby recruiting Fas‐associated death domain protein (FADD) and caspase‐8 for activation of caspase‐8 11.

Dasatinib, a tyrosine kinase inhibitor, has been approved for first‐line treatment of chronic myelogenous leukemia 12. Various ongoing studies are evaluating the effectiveness of dasatinib in different cancers 13, 14, 15, 16, but the mechanisms underlying the effect of dasatinib on GC progression are not yet clear. Therefore, in this study, we analyzed how dasatinib suppresses the growth of GC cells and interacts with other chemotherapeutic drugs. The results revealed that dasatinib sensitized TRAIL‐mediated apoptosis via DR5 induction in a CCAAT/enhancer‐binding protein homologous protein (CHOP)‐dependent manner, suggesting that the inductive effect of DR5 may be used to evaluate the efficiency of dasatinib and other drugs for chemotherapy.

## Materials and methods

### Cell culture

The GC cell lines NCI‐N87, SNU‐16, SNU‐5, Hs746T, KATO III and SNU‐1 were obtained from the American Type Culture Collection (Manassas, VA, USA). Dulbecco's modified Eagle's medium supplemented with 10% FBS (Invitrogen, Carlsbad, CA, USA) was used for cell culture. Dasatinib (Selleckchem, Houston, TX, USA) was diluted with dimethyl sulfoxide, and TRAIL (Sigma‐Aldrich, St Louis, MO, USA) was diluted with water.

### Quantitative real time polymerase chain reaction

Total RNA was extracted using TRIzol (Invitrogen) and reverse transcribed to cDNA. Thereafter, PCR was performed in triplicate, using a 20 μL reaction mixture containing gene‐specific primers and probes, and relevant procedures were also performed. DR5 mRNA expression was evaluated, using β‐actin expression as the internal reference. The results were analyzed using the $2^{-\Delta \Delta C_{t}}$ method. The primers are as follows. DR5: forward: 5′‐AAGACCCTTGTGCTCGTTGT‐3′, reverse: 5′‐AGGTGGACACAATCCCTCTG‐3′; β‐actin: forward: 5′‐GACCTGACACACTACCTCAT‐3′, reverse: 5′‐AGACAGCACTGTGTTGGCTA‐3′.

### Western blotting assay

Western blotting was performed in accordance with a previously reported method 17. The assay was performed using antibodies against the following proteins: FADD, cleaved caspase 3, cleaved caspase 8, CHOP (Cell Signaling Technology, Danvers, MA, USA), DR5, DR4, Bip, β‐actin, Fas, Mcl‐1, Bcl‐2 and Bcl‐X_L_ (BD Biosciences, San Jose, CA, USA).

### Detection of cell apoptosis

Nuclear staining with Hoechst 33258 was performed for apoptosis analysis 18. Subsequently, we performed propidium iodide staining. Caspase activity was measured using the SensoLyte Homogeneous AMC Caspase‐3/7 Assay Kit (AnaSpec, Fremont, CA, USA) as per the manufacturer's instructions.

### MTS assay

Cells were inoculated into a 96‐well plate at 1 × 10^4^ cells per well and incubated overnight, followed by treatment with dasatinib for 72 h. The MTS assay was performed in strict accordance with the MTS assay kit (Promega, Fitchburgh, WI, USA) instructions, followed by measurement of luminescence. This experiment was performed in triplicate.

### Cell transfection and gene knockdown

NCI‐N87 cells were transfected with small interfering RNA (siRNA) 24 h before dasatinib treatment. The control siRNA and siRNAs for CHOP (AAGACCCGCGCCGAGGUGAAG) and DR5 (AAGACCCUUGUGCUCGUUGUC) were purchased from Invitrogen. For stable transfection of NCI‐N87 cells, a short hairpin RNA (shRNA)‐expressing plasmid with a *FADD*‐targeting sequence (GCA GUC CUC UUA UUC UAA) or DR5‐targeting sequence (AAG ACC CUU GUG CUC GUU GUC) was used or an empty vector was used. Thereafter, the cells were incubated in a 96‐well plate with puromycin (4 μg·mL^−1^), followed by western blotting to detect the protein expression of the relevant clones.

### ChIP

ChIP was performed using the CHOP antibody (Cell signaling Technology) in accordance with the Chromatin Immunoprecipitation Assay Kit (Sigma‐Aldrich). PCR was performed using the corresponding primers for amplification of the targeted fragments of DR5.

### Xenograft experiments

Briefly, 5 × 10^6^ NCI‐N87 cells in 0.1 mL of PBS were implanted subcutaneously on the back of athymic nude female mice. After a week, mice were treated daily with dasatinib at 10 mg·kg^−1^ for 10 consecutive days by oral gavage. Tumor growth was monitored by calipers, and tumor volumes were calculated according to the formula 1/2 × length × width^2^. Mice were euthanized when tumors reached about 1.0 cm^3^ in size. Tumors were dissected and fixed in 10% formalin and embedded in paraffin. Terminal deoxynucleotidyl transferase‐mediated dUTP nick end labeling (TUNEL) and active caspase 3 immunostaining were performed on 5 μm paraffin‐embedded tumor sections by using an AlexaFluor 488‐conjugated secondary antibody (Invitrogen) for signal detection.

### Statistical analysis

The data was analyzed with graphpad prism v software (La Jolla, CA, USA). Student's *t*‐test was performed for comparisons, and *P* < 0.05 was considered to indicate statistical significance. The means ± standard deviation (SD) are displayed in the figures.

## Results

### Dasatinib decreased proliferation and induced apoptosis in GC cells

**Figure 1:**
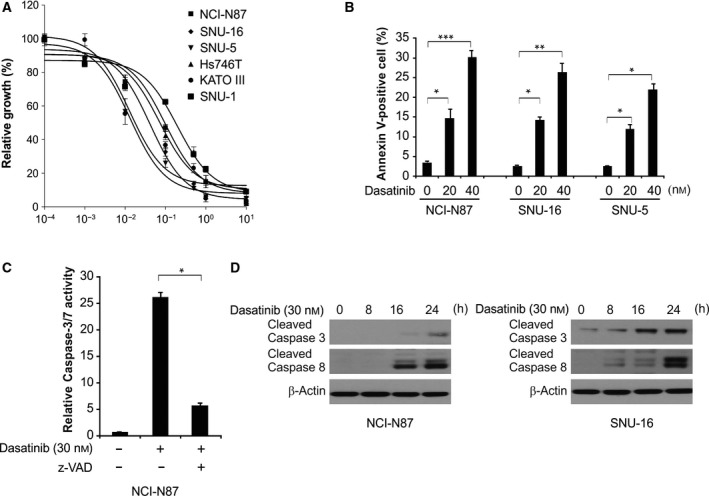
Dasatinib induces apoptosis in GC cells. (A) MTS assay for cell proliferation after 72 h of treatment with dasatinib in gradient concentrations. (B) Flow cytometry for apoptosis of cell lines after 24 h of treatment with dasatinib at indicated concentrations. (C) Fluorogenic analysis of caspase‐3/7 in NCI‐N87 cells after treatment of dasatinib with or without z‐VAD. (D) Western blotting analysis of cleaved caspase‐3/8 in cell lines after treatment with 30 nmol·L^−1^ dasatinib at indicated time points. ***P < 0.001, **P < 0.01, *P < 0.05.

To determine the efficacy of dasatinib in GC treatment, the cells were treated with dasatinib with an increasing concentration for 3 days, followed by detection of cell proliferation with the MTS assay. We found that GC cell proliferation significantly decreased (Fig. 1A). In the apoptosis assays, treatment with dasatinib at 20 or 40 nmol·L^−1^ increased the population of Annexin V‐positive cells in NCI‐N87, SNU‐16 and SNU‐5 cells (Fig. 1B). Furthermore, our findings showed that dasatinib induced the activation of caspases 3/7 in NCI‐N87 cells (Fig. 1C). The apoptotic response was also attenuated in the presence of carbobenzoxy‐valyl‐alanyl‐aspartyl‐[*O*‐methyl]‐fluoromethylketone (z‐VAD‐fmk), a pan‐caspase inhibitor (Fig. 1C), indicating that apoptosis induction depends on caspase activity. We detected cleaved forms of caspase‐3 and caspase‐8 in dasatinib‐treated NCI‐N87 and SNU‐16 cells for 24 h and found that dasatinib activates caspase‐3 and caspase‐8 in these cells (Fig. 1D). These data indicate that in the presence of dasatinib, GC cell proliferation decreased and caspase‐dependent apoptosis was induced.

### FADD and caspase‐8 are essential to dasatinib‐induced cell apoptosis in GC

**Figure 2:**
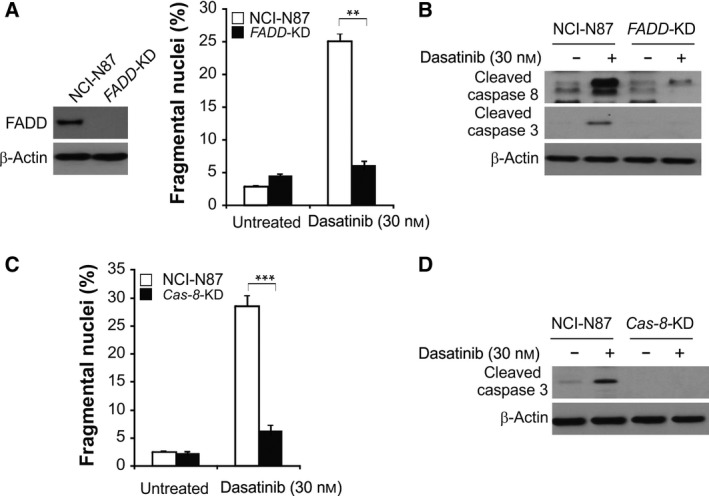
Caspase 8 and FADD are required for dasatinib‐induced apoptosis. (A) Apoptosis analysis of parental and stable *FADD*‐knockdown (*FADD*‐KD) NCI‐N87 cells after treatment with 30 nmol·L^−1^ dasatinib for 24 h. (B) Western blotting analysis of cleaved caspase‐3/8 in parental and *FADD*‐KD NCI‐N87 cells after treatment with 30 nmol·L^−1^ dasatinib for 24 h. (C) Apoptosis analysis of parental and stable *Cas‐8*‐KD NCI‐N87 cells after treatment with 30 nmol·L^−1^ dasatinib for 24 h. (D) Western blotting analysis of cleaved caspase‐3/8 in parental and *Cas‐8*‐KD NCI‐N87 cells after treatment with 30 nmol·L^−1^ dasatinib for 24 h. ***P < 0.001, **P < 0.01.

Since the above data showed that dasatinib can activate caspase‐8, we compared the inductive effects of dasatinib on apoptosis in parental cells and *FADD* stable knockdown (*FADD*‐KD) NCI‐N87 cells. Dasatinib‐induced apoptosis markedly decreased in *FADD*‐KD cells (Fig. 2A,B). Next, we investigated whether caspase‐8 is essential for dasatinib‐induced apoptosis. We treated parental cells and caspase‐8 stable knockdown (*Casp‐8*‐KD) NCI‐N87 cells with dasatinib and analyzed apoptosis by nuclear staining. Compared to parental cells, *Casp‐8*‐KD cells showed remarkably lesser dasatinib‐induced apoptosis (Fig. 2C,D), indicating that dasatinib‐induced apoptosis is dependent on caspase‐8. The above results show that the absence of FADD can lead to a decrease in dasatinib‐induced apoptosis in cancer cells, suggesting that the activated extrinsic apoptotic pathway is key to dasatinib‐induced apoptosis.

### Dasatinib upregulated DR5 expression in GC cells

**Figure 3:**
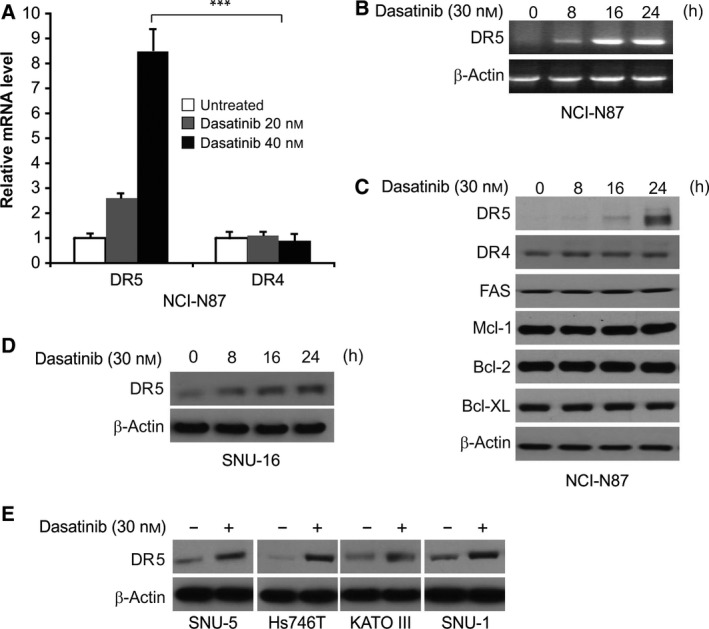
Dasatinib increases DR5 expression in GC cells. (A) RT‐PCR analysis of *DR5* mRNA expression in NCI‐N87 cells after treatment with dasatinib for 24 h at indicated concentrations. (B) Analysis of *DR5* mRNA level through gel electrophoresis in NCI‐N87 cells after treatment with 30 nmol·L^−1^ dasatinib at indicated time points with β‐actin for normalization. (C) Western blotting analysis of indicated proteins in NCI‐N87 cells after treatment with 30 nmol·L^−1^ dasatinib at indicated time points. (D) Western blotting analysis of DR5 in SNU‐16 cells after treatment with dasatinib for 24 h at indicated concentrations. (E) Western blotting of DR5 expression in GC cells treated with 30 nmol·L^−1^ dasatinib for 24 h. ***P < 0.001.

We investigated the mechanism underlying the activation of extrinsic apoptosis by dasatinib. We detected DR4 and DR5 expression in NCI‐N87 cells treated with dasatinib. DR4 and DR5, as the DRs on the cell surface, are important for the activation of the extrinsic apoptotic signaling pathway through FADD. Dasatinib markedly induced DR5, but not DR4, protein and mRNA expression in a dose‐ and time‐dependent manner in NCI‐N87 and other GC cells (Fig. 3A–E). However, FAS protein expression did not vary on treatment with dasatinib, and similar results were obtained for the protein expression of Bcl‐2 family members, including Mcl‐1, Bcl‐2 and Bcl‐XL (Fig. 3C). The above results indicate that dasatinib greatly increases the expression of DR5, but not DR4, in GC.

### Dasatinib enhanced TRAIL‐induced cell apoptosis

**Figure 4:**
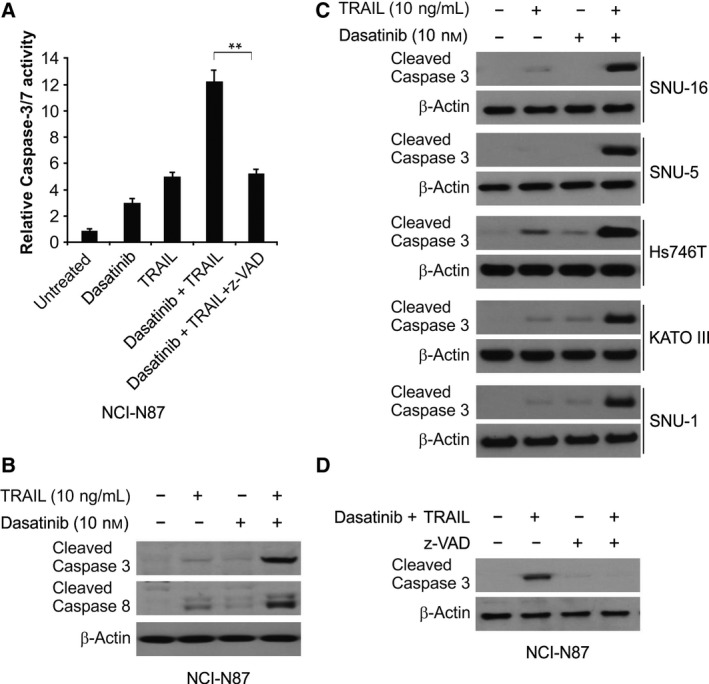
Dasatinib sensitizes TRAIL‐mediated apoptosis. (A) Apoptosis analysis of NCI‐N87 cells after treatment with 10 nmol·L^−1^ dasatinib alone, 10 ng·mL^−1^ TRAIL alone or their combination with or without 10 μmol·L^−1^ z‐VAD for 24 h. (B) Western blotting analysis for cleaved caspase‐3/8 in NCI‐N87 cells after treatment with 10 nmol·L^−1^ dasatinib alone, 10 ng·mL ^−1^ TRAIL alone or their combination for 24 h. (C) Western blotting analysis for cleaved caspase‐3 in SNU‐16, SNU‐5, Hs746T, KATO III and SNU‐1 cells after treatment with 10 nmol·L^−1^ dasatinib, 10 ng·mL ^−1^ TRAIL or their combination for 24 h. (D) Western blotting analysis for cleaved caspase‐3 in NCI‐N87 cells after treatment with the combination of 10 nmol·L^−1^ dasatinib and 10 ng·mL ^−1^ TRAIL with or without 10 μmol·L^−1^ z‐VAD for 24 h. **P < 0.01.

We investigated the role of dasatinib in sensitizing TRAIL‐mediated apoptosis in GC. We determined the effects of dasatinib, TRAIL and a combination of both on cell death. The inductive effect of a combination of dasatinib and TRAIL on apoptosis was more evident than that obtained on treatment with dasatinib or TRAIL alone (Fig. 4A). In addition, after cells were pretreated with z‐VAD‐fmk, the apoptotic response was also found to be attenuated (Fig. 4A). Consistent with this, a combination of dasatinib and TRAIL was also more effective than treatment with either used alone in increasing cleaved caspase‐3 and caspase‐8 in NCI‐N87 cells, as well as in increasing cleaved caspase‐3 in other GC cells (Fig. 4B,C). After pretreatment with z‐VAD‐fmk, the ability of the dasatinib and TRAIL combination to induce caspase‐3 activation was inhibited (Fig. 4D). The findings indicated that dasatinib in combination with TRAIL can enhance cell apoptosis induction in GC.

### Increased DR5 expression facilitated dasatinib‐induced apoptosis

**Figure 5:**
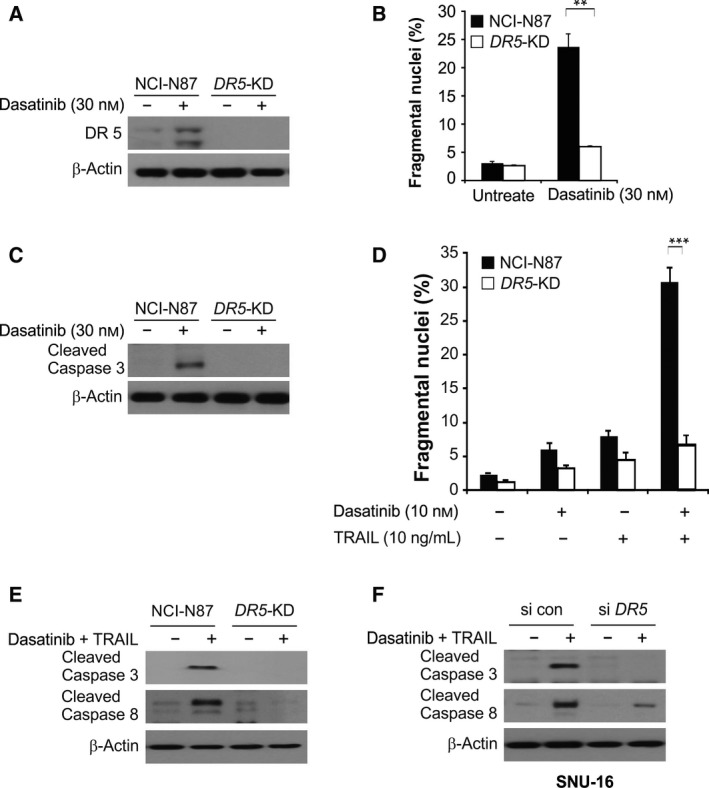
DR5 is required for dasatinib‐induced apoptosis. (A) Western blotting analysis of DR5 protein expression in parental and stable *DR5*‐KD NCI‐N87 cells after treatment with 30 nmol·L^−1^ dasatinib for 24 h. (B) Apoptosis analysis of parental and *DR5*‐KD NCI‐N87 cells after treatment with 30 nmol·L^−1^ dasatinib for 24 h. (C) Western blotting analysis of cleaved caspase‐3 in parental and *DR5*‐KD NCI‐N87 cells after treatment with 30 nmol·L^−1^ dasatinib for 24 h. (D) Apoptosis analysis of parental and *DR5*‐KD NCI‐N87 cells after treatment with 10 nmol·L^−1^ dasatinib for 24 h, 10 ng·mL ^−1^ TRAIL or their combination for 24 h. (E) Western blotting analysis of cleaved caspase‐3/8 in parental and *DR5*‐KD NCI‐N87 cells after treatment with the combination of 10 nmol·L^−1^ dasatinib and 10 ng·mL ^−1^ TRAIL with or without 10 μmol·L^−1^ z‐VAD for 24 h. (F) Western blotting analysis of cleaved caspase‐3/8 in SNU‐16 cells after transfection with si control or si *DR5* for 24 h, and treatment with the combination of 10 nmol·L^−1^ dasatinib and 10 ng·mL ^−1^ TRAIL with or without 10 μmol·L^−1^ z‐VAD for 24 h. ***P < 0.001, **P < 0.01.

The effects of dasatinib on cell apoptosis and caspase activation were compared between the parental cells and DR5‐knockdown (*DR5‐*KD) NCI‐N87 cells to determine the role of DR5 expression in GC cell apoptosis (Fig. 5A). We found that dasatinib‐induced apoptosis remarkably decreased in *DR5*‐KD cells (Fig. 5B). Moreover, dasatinib strongly induced caspase‐3 cleavage in parental NCI‐N87 cells, but was blocked in *DR5*‐KD cells (Fig. 5C). To clarify whether dasatinib can affect TRAIL‐induced cell apoptosis through increased DR5 expression, we treated the cells with a combination of dasatinib and TRAIL; in parental cells treated with the combination, apoptosis and caspase‐3 and caspase‐8 cleavage significantly increased (Fig. 5D,E). Knockdown of DR5 by siRNA significantly suppressed caspase‐3 and caspase‐8 cleavage in SNU‐16 cells (Fig. 5F). These findings suggest that DR5 expression is upregulated in the presence of dasatinib, which can further enhance TRAIL‐induced cell apoptosis.

### Dasatinib induced increase in DR5 expression, which was mediated by CHOP

**Figure 6:**
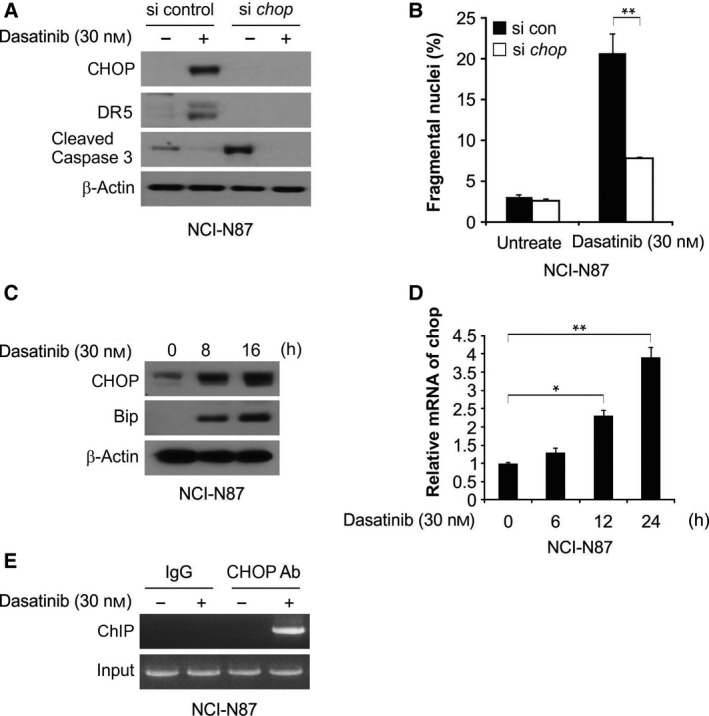
Dasatinib induces CHOP‐mediated DR5 expression. (A) Western blotting analysis of CHOP, DR5 and cleaved caspase‐3 in NCI‐N87 cells after transfection with si control or si *chop* for 24 h, and treatment with 30 nmol·L^−1^ dasatinib for 24 h. (B) Apoptosis analysis of NCI‐N87 cells after transfection with si control or si *chop* for 24 h, and treatment with 30 nmol·L^−1^ dasatinib for 24 h. (C) Western blotting analysis of CHOP and Bip in NCI‐N87 cells after treatment with 30 nmol·L^−1^ dasatinib at indicated time points. (D) The level of mRNA in NCI‐N87 cells after treatment with 30 nmol·L^−1^ dasatinib at indicated time points with β‐actin for normalization. (E) ChIP was carried out with anti‐CHOP antibody on NCI‐N87 cells after treatment with dasatinib for 12 h, while ChIP with the control IgG was used as a control. PCR was performed with the corresponding primers. **P < 0.01, *P < 0.05.

Given the involvement of CHOP in DR5 upregulation and the contribution to sensitization development in TRAIL‐mediated apoptosis, we investigated whether dasatinib can affect CHOP‐dependent DR5 expression in GC, as well as the mechanism underlying dasatinib/TRAIL‐induced apoptosis. CHOP knockdown by siRNA attenuated DR5 induction, as well as apoptosis and caspase activation by dasatinib (Fig. 6A,B). Analysis of the effect of dasatinib on CHOP expression showed that, in NCI‐N87 cells treated with dasatinib, significant increases in CHOP mRNA and protein expression were induced in a time‐dependent manner (Fig. 6C,D). We also investigated whether *DR5* transcription is directly activated by CHOP. ChIP results showed CHOP recruitment in the genomic region containing the CHOP‐binding sites, following dasatinib treatment (Fig. 6E). Thus, the above results suggest that CHOP, through binding with the *DR5* promoter, can initiate the activation of DR5 transcription in response to dasatinib treatment.

### DR5 contributes to the antitumor activity of dasatinib *in vivo*

**Figure 7:**
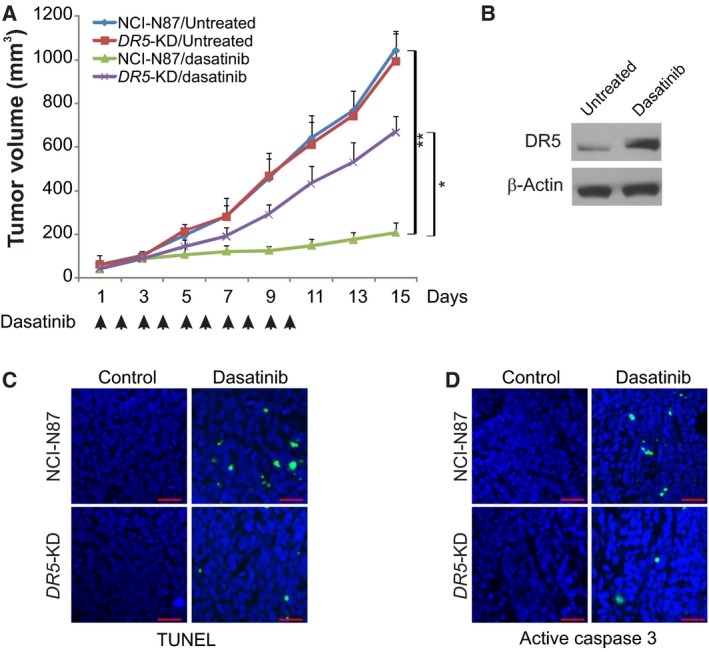
DR5 mediates the antitumor effects of dasatinib *in vivo*. (A) Nude mice were injected s.c. with 5 × 10^6^ parental and *DR5*‐KD NCI‐N87 cells. After 1 week, mice were treated with 10 mg·kg^−1^ dasatinib or buffer for 10 consecutive days. Tumor volume at indicated time points after treatment was calculated and plotted (*n* = 6 in each group). (B) Parental tumors were treated with 10 mg·kg^−1^ dasatinib or the control buffer as in (A) for 4 consecutive days. DR5 expression in the tumor was analyzed by western blotting. (C) Paraffin‐embedded sections of tumor tissues were analyzed by TUNEL staining. (D) Tissue sections from (C) were analyzed by active caspase 3 staining. Results of (A) are expressed as means ± SD of three independent experiments. **P < 0.01, *P < 0.05. Scale bars: 25 μm.

Next, we used a xenograft model to check DR5‐mediated antitumor effects of dasatinib *in vivo*. Nude mice were injected with parental and *DR5*‐KD NCI‐N87 cells were treated with 10 mg·kg^−1^ dasatinib or vehicle by oral gavage for 10 consecutive days. As shown in Fig. 7A, parental and *DR5*‐KD tumors were not significantly different in growth in the vehicle treatment groups. Dasatinib treatment suppressed the growth of parental tumors by 70–80%. However, compared with parental tumors, *DR5*‐KD tumors were insensitive to dasatinib treatment, indicating that loss of DR5 abrogated the antitumor activity of dasatinib. Furthermore, increased DR5 expression was detected in dasatinib‐treated xenograft tumors (Fig. 7B). TUNEL staining revealed significant apoptosis induction in tumor tissues from the dasatinib‐treated mice, but not the control mice. In contrast, apoptosis was barely detectable in the *DR5*‐KD tumors (Fig. 7C). Staining for active caspase 3 verified DR5‐dependent apoptosis in dasatinib‐treated tumors (Fig. 7D). Thus, the *in vivo* antitumor and apoptotic activity of dasatinib is DR5 dependent.

## Discussion

This study showed that dasatinib can effectively attenuate cell proliferation by inducing cell apoptosis in GC. In addition, our results showed that cells could escape cell death and caspase cleavage through the dasatinib‐induced extrinsic apoptotic pathway. Upregulation of DR5 expression induced by dasatinib and mediated by CHOP is necessary for the anticancer effects of dasatinib. Moreover, we found that, on combination treatment with dasatinib and TRAIL, DR5‐dependent cell apoptosis was induced, indicating that dasatinib can sensitize TRAIL‐induced cell apoptosis. Dasatinib promoted TRAIL‐mediated apoptosis via upregulation of CHOP‐dependent DR5 expression in GC. In the current study, DR5 induction can reflect sensitivity to dasatinib, indicating its significance in clinical application.

TRAIL, when used as an alternative anticancer agent, can induce cell apoptosis in various cancers but without any cytotoxic effects on normal cells; therefore, it has been considered as a promising novel target for anticancer treatment 19, 20, 21. However, resistance to TRAIL has been reported frequently in various cancers 22. Thus, agents that can increase the sensitivity of cancer cells to TRAIL are urgently required to improve the efficacy of TRAIL treatment. TRAIL mediates apoptosis through binding with DR5 and DR4, leading to generation of the death‐inducing signaling complex and binding with caspase 8 23, 24. In our current study, we found that dasatinib decreased cell proliferation and induced GC cell apoptosis associated with the activated extrinsic apoptotic pathway (e.g. FADD and caspase‐8). Moreover, we found that dasatinib upregulated DR5 expression in GC cells. Dasatinib enhanced cell apoptosis induced by TRAIL. A previous study showed that DR5 and DR4 expression is also critical for the sensitivity of breast cancer cells to TRAIL 25. Thus, DR5 is a critical link in the development of the antitumor effect of dasatinib in GC cells.

Targeting the TRAIL/DR5 extrinsic apoptotic pathway with agonist DR5 antibodies or recombinant TRAIL has become as an attractive cancer treatment strategy 26, 27. However, previous studies have shown that only DR5 agonistic antibodies may be effective 28, 29. Multiple phase I single agent studies with advanced tumors were completed using TRA‐8/CS‐1008, drozitumab, lexatumumab and conatumumab 30, 31, 32, 33. Despite there being several reports of stable disease with DR5‐targeting agonistic antibodies, only conatumumab showed modest activity in phase II and showed partial response in non‐small cell lung cancer patients in a single drug trial 34. Preliminary clinical studies of DR5‐targeting antibodies have shown rather low initial clinical activity 26. Additional work, such as antibody optimization, stratification of patients based on certain biomarkers, or in combination with other drugs to synergize antibody activity may be required to get better clinical results.

CHOP is upregulated by endoplasmic reticulum (ER) stress and is involved in ER‐mediated apoptosis; it is a transcription factor associated with DR5 upregulation 35, 36. Here, our results showed that increased DR5 expression facilitated dasatinib‐induced apoptosis. Dasatinib induced increase in DR5 expression, which was mediated by CHOP, indicating that dasatinib‐mediated CHOP upregulation is a key regulator of DR5 expression and that CHOP is essential for dasatinib‐induced apoptosis. Furthermore, some studies have reported that CHOP induces apoptosis through ER stress and that CHOP knockdown blocks cell apoptosis induced by various anticancer agents 37, 38. Under ER stress, CHOP expression is also facilitated, usually leading to an increase in the expression of apoptosis‐related factors, including DR5 39. Our study further showed that DR5 is required for dasatinib‐induced apoptosis. Dasatinib also synergized with TRAIL to induce significant apoptosis in GC cells via DR5. Our findings helped elucidate the effects of DR5 on the mediation of dasatinib‐induced apoptosis, enhancement of TRAIL‐induced apoptosis, and the mechanisms underlying dasatinib‐induced DR5 upregulation.

In conclusion, our results show that dasatinib has a potent inhibitory effect on GC cells. In addition, it sensitizes TRAIL‐mediated apoptosis through CHOP‐dependent DR5 upregulation. DR5 induction indicates sensitivity to dasatinib and sheds light on its potential clinical applications.

## Author contributions

XW and HL conceived and designed the project. XW, QX, LW and BW performed the experiments. XW and HL wrote the paper.
